# Competing Heterogeneities in Vaccine Effectiveness Estimation

**DOI:** 10.3390/vaccines11081312

**Published:** 2023-08-01

**Authors:** Ariel Nikas, Hasan Ahmed, Veronika I. Zarnitsyna

**Affiliations:** 1 Department of Microbiology and Immunology, Emory University School of Medicine, Atlanta, GA 30322, USA; 2 Department of Biology, Emory University, Atlanta, GA 30322, USA; hasan.r.ahmed@emory.edu

**Keywords:** vaccine effectiveness, heterogeneity, frailty, antibody waning by power law, selection, vaccine efficacy, survival analysis

## Abstract

Understanding the waning of vaccine-induced protection is important for both immunology and public health. Population heterogeneities in underlying (pre-vaccination) susceptibility and vaccine response can cause measured vaccine effectiveness (mVE) to change over time, even in the absence of pathogen evolution and any actual waning of immune responses. We use multi-scale agent-based models parameterized using epidemiological and immunological data, to investigate the effect of these heterogeneities on mVE as measured by the hazard ratio. Based on our previous work, we consider the waning of antibodies according to a power law and link it to protection in two ways: (1) motivated by correlates of risk data and (2) using a within-host model of stochastic viral extinction. The effect of the heterogeneities is given by concise and understandable formulas, one of which is essentially a generalization of Fisher’s fundamental theorem of natural selection to include higher derivatives. Heterogeneity in underlying susceptibility accelerates apparent waning, whereas heterogeneity in vaccine response slows down apparent waning. Our models suggest that heterogeneity in underlying susceptibility is likely to dominate. However, heterogeneity in vaccine response offsets <10% to >100% (median of 29%) of this effect in our simulations. Our study suggests heterogeneity is more likely to ‘bias’ mVE downwards towards the faster waning of immunity but a subtle bias in the opposite direction is also plausible.

## 1. Introduction

Providing accurate estimates of vaccine-induced protection is essential in guiding public health policy. However, many factors complicate our ability to estimate population-level vaccine effectiveness (VE) such as prior immunity, underlying health risks, timing of vaccination, inconsistent hazards in different locations, and other confounders. Further adding to uncertainty is the common presence of observed waning which may reflect the actual waning of immune responses, introduction of different strains, or may be an artifact coming from heterogeneity among individuals. Many studies report the fast, intraseasonal waning of vaccine-induced protection, particularly for viruses such as influenza and SARS-CoV-2 [1,2,3]; however, various effects can bias this conclusion. The depletion of susceptible individuals (also called the *frailty effect* in biostatistics) can bias estimates [4,5]. Heterogeneity in exposure risk, even if exactly the same in the vaccinated and unvaccinated groups, tends to bias the vaccine effectiveness estimates downwards potentially leading to spurious claims of waning [6,7]. If natural immunity is not taken into account, merely having a “leaky” vaccine (i.e., a vaccine that provides partial protection) can bias estimates downwards [5,7]. This complicates the estimation of the actual waning of vaccine-induced protection, which is expected to occur as many correlates of protection, e.g., neutralizing antibodies, have been shown to decrease over time [8,9,10,11].

Although discussing heterogeneity in an abstract mathematical sense is common [4,5,6,7,12] (usually assuming heterogeneity to follow known statistical distributions), understanding this more concretely, along with certain nuances, is also important. Individual-to-individual heterogeneity in the risk of acquiring infection can conceptually be decomposed into two factors: heterogeneity in underlying susceptibility and heterogeneity in vaccine-induced protection. The former can arise because of behavioral pattern differences affecting the amount of contact with potentially infectious individuals (e.g., frequently riding the subway may increase such contacts), whereas the latter can arise from a variability in antibody and T-cell responses [11,13,14,15]. We assume that these behavioral patterns persist over the relevant time scale (see also the Discussion section on regression towards the mean). While our results apply to heterogeneity in underlying susceptibility and heterogeneity in vaccine-induced protection from all sources, we specifically focus on two sources—the amount of contact with potentially infectious individuals, which contributes to underlying susceptibility, and antibody level, which contributes to vaccine-induced protection.

In this paper, we focus on the hazard ratio as the measure of vaccine effectiveness, as the hazard ratio corresponds to relative risk at a particular moment in time. To determine the direction and magnitude of bias, we simulate an epidemic in a population under various frailty and vaccine protection distributions in the absence and presence of waning and evaluate the interplay between these factors. Commonly, hazard ratios are estimated with the Cox proportional hazards model and there are several standard extensions utilized in real-world studies [16,17,18,19,20,21,22,23]. While Cox proportional hazard models were not intended to be time-varying, several approaches exist in order to make it applicable for measuring waning vaccine effectiveness. We utilize time category–vaccine interactions (henceforth referred to as TVI) which, in contrast to the commonly used Cox method utilizing the scaled Schoenfeld residuals, should accurately measure the hazard ratios even in the presence of extreme (observed) waning [24].

If vaccine effectiveness is assessed via the hazard ratio and the outcome of interest is the first infection post-vaccination, heterogeneity in baseline (pre-vaccination) susceptibility causes measured vaccine effectiveness (mVE) to decline over time, whereas heterogeneity in response to vaccination causes mVE to increase over time. Hence, any apparent change in mVE may reflect any combination of the selection on these heterogeneities in addition to the biological processes of pathogen evolution and waning of immune responses. In this paper, we first illustrate the problem using standard statistical distributions for both of the heterogeneities. We then provide concise formulas that give the net effect of these heterogeneities on the hazard rates and hazard ratio. Next, using parameter values based on epidemiological and immunological data incorporating the waning of antibodies, we use agent-based simulations to investigate the magnitude of these opposing effects. Our models suggest that the larger effect is from heterogeneity in baseline susceptibility but that heterogeneity in vaccine response may offset a substantial fraction of that effect. This exacerbation of observed waning may explain the negative VE reported in some studies [25,26].

## 2. Methods

We consider an agent-based model of acute viral infection with a constant background force of infection where we introduce heterogeneity in individual infection risk, heterogeneity in vaccine-induced protection, or both. For most scenarios to be described, vaccine protection follows the “leaky” model, wherein each vaccinated individual experiences a certain percent reduction in risk. Additionally, we model 40% out of a cohort of 100,000 to be vaccinated, in line with the CDC estimate for influenza vaccine coverage [27]. Since we model an acute viral infection, we assume sterilizing immunity upon infection for the remainder of the one-year time period considered. All simulations were run in *Julia* version 1.3.1 (Julia Project, github.com/JuliaLang) and statistical analysis was completed in *R* software version 4.2.1 (R Foundation, Vienna, Austria).

For heterogeneity in underlying risk (risk in the absence of vaccination), we select a daily risk rate for both the vaccinated and unvaccinated groups from a single gamma distribution. For heterogeneity in vaccine protection, we select vaccinated individuals’ protection from a variety of distributions including beta distributions, in contrast to leaky, homogeneous vaccination where all individuals’ protection would be the same. To establish a comparison, we use the mean vaccine efficacy in the context of no epidemic, VE_NE_, which thereby removes the effect of selection. We then calculate vaccine effectiveness using a time category–vaccine interaction (TVI) as the independent variable of the Cox proportional hazards model in order to find a time-varying estimate of protection. The TVI method has been shown to behave accurately in circumstances where waning occurs rapidly [24].

## 3. Results

### 3.1. Underlying Susceptibility to Infection (Frailty) Distribution

**Figure 1:**
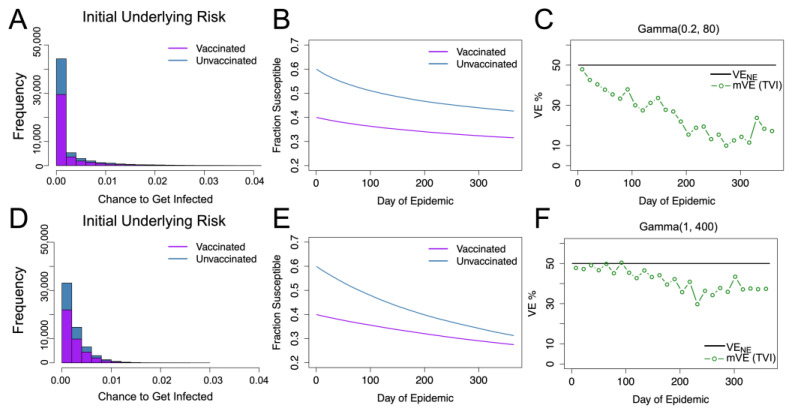
Gamma distributed underlying risk with vaccine protection (VE) homogeneous at 50% protection. For underlying risk described by Gamma (0.2, 80), the distribution of the unvaccinated and vaccinated population’s daily risk is given in blue and purple as shown in (**A**). (**B**) shows the fractions of the susceptible individuals in each group. (**C**) shows the estimated vaccine effectiveness (mVE), which drops markedly below the given level we expect of the leaky vaccine (black), decreasing to 17% from the original 50%. (**D**–**F**) display the same results for Gamma (1, 400). As seen in (**F**), the estimated vaccine effectiveness is below the true value but is not as severe as (**C**), only decreasing to 38.5%.

When considering only heterogeneity in underlying frailty, the given gamma distributions (parameterized as Gamma(*α_γ_*, *β_γ_*) where *α_γ_*/*β_γ_* is the mean and *α_γ_*^−0.5^ is the coefficient of variation (CoV)) can produce the appearance of waning vaccine effectiveness, though as *α_γ_* increases this effect lessens. This appearance of waning corresponds to what many statistical studies have posited would occur, and is termed the “frailty effect” or “frailty phenomena” [28,29,30,31]; in epidemiological studies, this is sometimes alternatively called “survivor bias” or the “depletion of susceptibles” effect. Some studies have also simulated this effect but have not compared the qualitative effect of different distributions [5,6]. In Figure 1, we show how different gamma distributions with the same mean can cause differing amounts of perceived waning (waning in mVE) when the true vaccine protection is in fact constant and leaky.

### 3.2. Vaccine Efficacy Distribution

**Figure 2:**
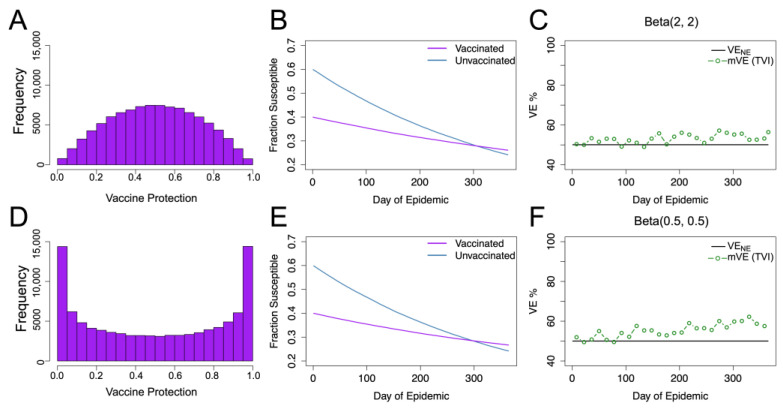
Beta distributed vaccine protection with homogeneous underlying risk. (**A**–**C**) give the results for vaccine protection distributed Beta(2, 2), where (**A**) displays the resultant distribution in the vaccinated population, (**B**) shows the change in susceptible populations (as a fraction of total population) over time, and (**C**) shows the vaccine effectiveness (mVE) estimate which increased by 6%. Keeping the mean the same but changing the distribution shape, as seen in (**D**), to Beta(0.5, 0.5), we, likewise, see similar infection dynamics (**E**) but a higher mVE (**F**), increasing by approximately 10%. Here, VE_NE_ is the vaccine efficacy if there were no epidemic, and it remains constant at 50%.

In simulated studies, two modes of vaccine efficacy are often compared: “leaky” vaccination, where protection is incomplete but reduces each individual’s chance to become infected by a specified amount (e.g., 50%), or “all-or-nothing” vaccination where a fraction of individuals receive complete protection from the vaccine and others receive no protection whatsoever. A limited number of studies have also considered normal-like distributions for vaccine protection [7]. We consider two main beta distributions parameterized by Beta(*α_β_*, *β_β_*) where *α_β_*/(*α_β_* + *β_β_*) is the mean (held here at 0.5): the normal-like Beta(2, 2) and the U-shaped Beta(0.5, 0.5). These distributions, as well as their resultant dynamics and mVE estimates, are compared in Figure 2, which shows that for both of these cases, distributions in vaccine protection bias VE estimates upwards. Non-symmetric vaccine protection distributions were also tested (see Figure S1 in Supplementary Materials) but did not change the direction of bias, continuing to show increase in mVE.

As seen in Figure 1 and Figure 2, singly, the two types of heterogeneity appear to contribute in opposite directions; beta distributed heterogeneity in vaccine protection tends to skew the estimate upwards while gamma distributed heterogeneity in underlying risk tends to skew the estimates downwards and to a greater extent. As these effects compete when combined, we constructed a predictor to determine which direction, if any, the competing distributions change mVE away from VE_NE_.

### 3.3. Effect of Selection on Heterogeneities

Assuming hazard rates for a given individual are not time-varying and ignoring stochasticity, $\bar{r}$, the average hazard rate in not-yet-infected individuals is given by the following equation:

(1) $$\bar{r}\left(t\right)=\frac{\int f\left(r\right)e^{-rt}r\;dr}{\int f\left(r\right)e^{-rt}\;dr}.$$

where $f\left(r\right)$ is the probability density function for the hazard rates at time 0; we allow $f\left(r\right)$ to be a generalized function (e.g., a delta function) so the formula also applies to discrete probability distributions. Let R denote the random variable for $f\left(r\right).$ Let $M\left(t\right)$ and $K\left(t\right)$ be the moment-generating and cumulant-generating functions of −R, respectively. Since the denominator of Equation (1) is $M\left(t\right)$ and the numerator is ${-M}^{\prime }\left(t\right)$ and $K\left(t\right)=\ln\left(M\left(t\right)\right)$, we obtain the following relation:

(2) $$-\bar{r}\left(t\right)=\frac{M^{\prime }\left(t\right)}{M\left(t\right)}=K^{\prime }\left(t\right).$$

Hence, the first derivative of $-\bar{r}\left(t\right)$ is the second cumulant (i.e., the variance) of −R, the second derivative of $-\bar{r}\left(t\right)$ is the third cumulant of −R (related to the skewness), the third derivative is the fourth cumulant (related to excess kurtosis), and so on. Since −R can be viewed as fitness, the above is essentially equivalent to a generalization of Fisher’s fundamental theorem of natural selection, according to which the *n*-th derivative of mean fitness over time is the *n* + 1 cumulant [32,33].

Since in most cases the force of infection is not constant, we further extended this relation between the hazard rates and cumulants. If we let $r_{i}=FOI\left(t\right)\cdot q_{i}$ where $r_{i}$ is an individual *i*’s hazard rate, *FOI* is the force of infection at time *t*, and *q_i_* is the individual’s relative hazard, we can recover the above relation in terms of a transformation of time $s=\int_{0}^{t}FOI\left(\tau \right)d\tau$,

(3) $$-\bar{r}\left(s\right)=\frac{M_{2}^{\prime }\left(s\right)}{M_{2}\left(s\right)}=K_{2}^{\prime }\left(s\right)$$

where $K_{2}$ is the cumulant generating function for the distribution of *−q_i_*.

We now consider the hazard ratio comparing vaccinated to unvaccinated individuals, $HR\left(t\right)=\bar{r_{v}}/\bar{r_{u}}$, where $\bar{r_{v}}$ is the average hazard rate in not-yet-infected vaccinated individuals and $\bar{r_{u}}$ is the analogous for unvaccinated individuals. To find the rate of change of the hazard ratio and recalling that the first derivative of the mean hazard rate is the second cumulant (i.e., variance) of the hazard rates, we apply the quotient rule (or the quotient and chain rules for the case of the time-varying *FOI*), which yields the following equation:

(4) $$\frac{d}{dt}\left[HR\right]=\frac{-\sigma_{v}^{2}\bar{r_{u}}+\sigma_{u}^{2}\bar{r_{v}}}{\bar{r_{u}^{2}}},$$

where $\sigma_{v}^{2}$ is the variance of the vaccinated group’s hazard rates and $\sigma_{u}^{2}$ is the variance of the unvaccinated group’s hazard rates.

If at time *t* = 0 the underlying risk is distributed Gamma(*α_γ_*, *β_γ_*) and vaccine protection is distributed Beta(*α_β_*, *β_β_*), and the two are independent, $\alpha_{\beta }+\beta_{\beta }-\alpha_{\gamma }$ determines the direction in which these heterogeneities affect *HR*(*t*) as

(5) $$\mathrm{sign}\left[HR^{\prime }\left(0\right)\right]=\mathrm{sign}\left(\alpha_{\beta }+\beta_{\beta }-\alpha_{\gamma }\right).$$

Hence, even in this simple scenario, heterogeneity can cause either an increase or decrease in mVE.

### 3.4. Vaccine Effectiveness under Competing Heterogeneities

**Figure 3:**
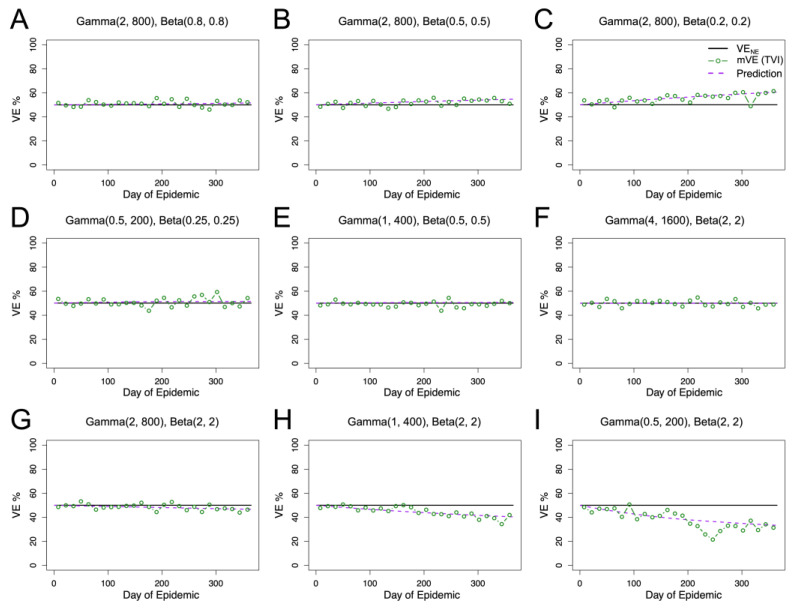
Competing heterogeneities allow for diverse outcomes in mVE. Per Equation (5), we predicted an increase in observed vaccine effectiveness in (**A**–**C**), no change in (**D**–**F**), and a decrease in (**G**–**I**). For all panels, our predicted value (purple dashed) closely matches the mVE (green). Given that an individual’s vaccine protection is constant for these simulations (black), this reiterates the difficulty in interpreting changes in mVE.

We considered the interplay of heterogeneities in both underlying frailty and vaccine protection and compared vaccine effectiveness estimates to our predicted value based on Equation (4). We found that overall estimates tended to oscillate around the predicted value (purple dashed), as seen in Figure 3. Here, we found that, depending on the underlying distributions, the mVE can increase, decrease, or remain steady around VE_NE_, the vaccine efficacy under no epidemic. Likewise, the extent of observed change was dependent on the interplay of both distributions, with some estimated VE values changing a negligible amount and others shown here changing greater than 20% compared to the VE_NE_ value. Furthermore, for all of the combinations shown in Figure 3, we maintained a VE_NE_ of 50%, but the initial protection level given also mediates how far from VE_NE_ a given distribution can change, as seen in Figure S2 in Supplementary Materials.

Even larger changes in mVE can be found by either extending the study period, allowing for more individuals to get infected and thus contributing to the over- or underestimation, or by considering a more extreme distribution. Additionally, it is theoretically possible to generate non-monotonic behavior, as shown in Figure S3 in Supplementary Materials, where mVE can go both up and down relative to VE_NE._

### 3.5. Modeling Waning Protection

Without direct challenge studies, estimating heterogeneity in vaccine effectiveness can be fraught. However, many studies use antibody titers as a correlate of protection, including those for SARS-CoV-2 [34,35,36]. Using data on waning SARS-CoV-2 antibodies, we created a distribution for initial protection in a population that then wanes over time. We modeled the waning of antibodies as a power law of the form

(6) $$Ab=C{\left[\left(t+41\right)/42\right]}^{-1}$$

in line with our previous studies [9,10,11]. The exponent −1 corresponds to relatively fast waning. Here, *C* for each individual is drawn from a log normal distribution with a standard deviation of 0.75–1.5 natural log in line with [11,13,14]. In previous studies, we analyzed antibodies and waning starting at day 42 post-infection or vaccination. We assumed here that all individuals in the vaccinated group were fully vaccinated before the study began. After this, we correlated an individual’s antibody level to their individual VE using

(7) $$1-VE=\min\left[Ab^{-1/2},1\right],$$

where the antibody-to-VE conversion exponent was based on an adjustment to the relationships given in [14] for HAI titers and the risk of infection, with exponents of approximately −0.35. Because HAI titer is only one component of the antibody response, we slightly increased the strength of the relationship and used −0.5. We call this the *risk-correlate model*.

We also considered a different relationship between the VE and antibody based on a within-host stochastic extinction model

(8) $$1-VE=\max\left[\frac{1-exp\left(\frac{m}{R_{0}}-\frac{m\cdot a}{a+k\cdot \mathrm{Ab}}\right)}{1-exp\left(\frac{m}{R_{0}}-m\right)},0\right],$$

where *a* = 10 is the death rate of virions, *R*_0_ = 10 is the basic reproductive number at the between-cell level [37], k⋅Ab represents the scaled level of antibodies, and *m* = 0.5 is the product of the number of viral particles per inoculum and a virion’s probability of successfully infecting a cell in the absence of antibodies (see Supplementary Materials for the derivation and additional details). This relationship gives qualitatively similar results to the risk-correlate model.

Antibody is scaled to give an approximately 90% initial vaccine protection in the population when the standard deviation for the natural log of the antibody is equal to 1, again in line with [11,13,14]; inherently, as standard deviation is varied, this causes the initial average of vaccine-induced protection in the population to vary slightly. This distribution replaces the beta distributions used in Section 3.4 for vaccine protection, while the underlying frailty in both groups continues to be modeled with gamma distributions, with the CoV based on a study [38] that estimated a CoV of 0.7–1.5 (mean of 0.9) based on contact surveys of a very short duration (e.g., two days) and a CoV of 0.3–0.9 (mean of 0.5) based on contact surveys when aggregating by 1-year age categories. As elaborated by [38], the former is likely an overestimate whereas the latter is likely an underestimate; hence, we considered a CoV of 0.5–1.

**Figure 4:**
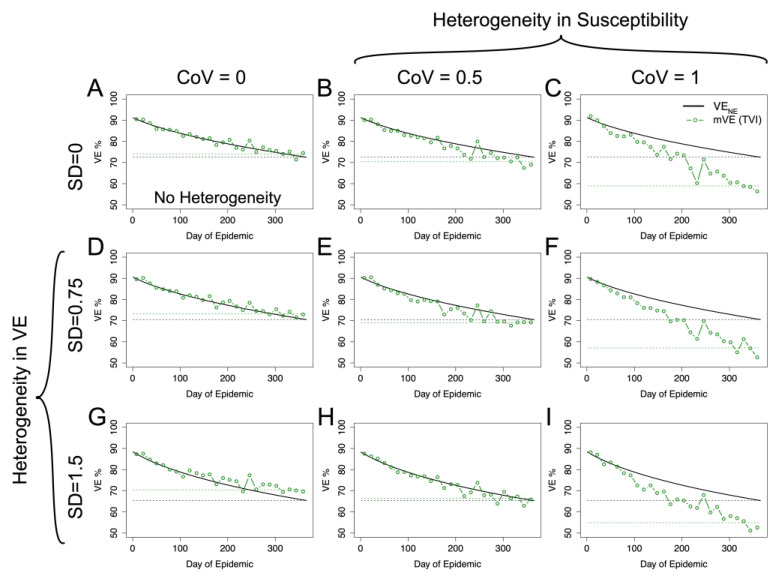
mVE for plausible acute infectious disease parameters is likely to underestimate VE_NE_ but not necessarily in all circumstances. Under no heterogeneities, as in (**A**), mVE is extremely close to VE_NE_; however, the introduction of heterogeneity biases the estimate. In the first column, showing simulations lacking heterogeneity in underlying susceptibility, heterogeneity in antibody biases the estimate upwards (**D**,**G**). In the first row, without any heterogeneity in antibody, the bias is downwards (**B**,**C**). With both, the underlying heterogeneity in susceptibility outcompetes heterogeneity in VE and leads to an underestimate relative to VE_NE_ (**E**,**F**,**I**), though not as extreme as would be seen if a vaccine was purely homogeneously leaky, except in (**H**) where the two effects approximately cancel each other out. SD indicates the standard deviation of antibody (at a given time) in natural logs; higher SD in antibody translates to higher variability in vaccine protection via Equation (6). CoV indicates the coefficient of variation in underlying susceptibility at the beginning of the simulation. The black dashed line shows the final VE_NE_ level reached in the population, while the green dashed line shows the final mVE as a mean of the last three estimated points.

Using the risk-correlate model, Figure 4 compares a simulation without any heterogeneity (Figure 4A) to simulations with just heterogeneity in (antibody-induced) vaccine protection or underlying frailty and simulations, with heterogeneity in both protection and underlying frailty, where underlying frailty is characterized by the coefficient of variation (CoV) of the gamma distribution where CoV = $1/\sqrt{\alpha_{\gamma }}$, and the mean of the gamma distributions are held the same as the previous figure. Recapitulating the earlier simulations, heterogeneity in vaccine protection results in an increase relative to VE_NE_. However, heterogeneity in underlying frailty overwhelms this positive trend and causes VE estimates to be underestimated. Similar qualitative results are also given using the unadjusted power law exponent estimated from the HAI titers (a likely underestimate) as shown in the Supplementary Materials.

**Figure 5:**
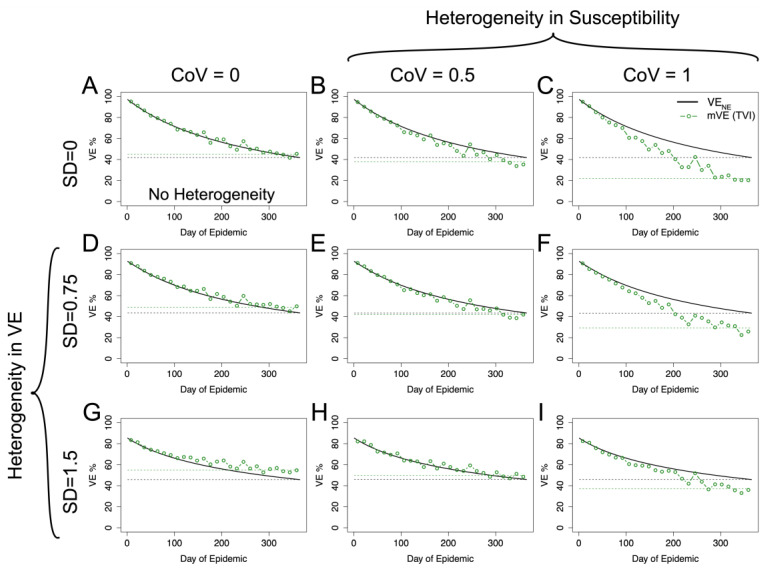
Within-host stochastic model. Utilizing the within-host stochastic model (Equation (8)) for antibody-mediated, vaccine-induced protection yields similar results to the risk-correlate model (Equation (7)). As expected, increasing heterogeneity in underlying susceptibility (moving left to right) pushes mVE downwards while increasing heterogeneity in vaccine-induced protection (moving top to bottom) pushes mVE upwards. When these effects are mixed, as in (**E**,**F**,**H**,**I**), the heterogeneities compete. (**A**) shows the no heterogeneity case, (**B**,**C**) heterogeneity in underlying susceptibility only, and (**D**,**G**) heterogeneity in vaccine-induced protection only. The black dashed line shows the final VE_NE_ level reached in the population, while the green dashed line shows the final mVE as a mean of the last three estimated points.

Using the within-host stochastic model given by Equation (8) yields similar results, as seen in Figure 5, to the risk-correlate model. For both models, the degree of the over- or underestimation (relative to VE_NE_) at the end of the season is given in Table S1 in Supplementary Materials. Again, for plausible acute infectious disease parameters, mVE tends to be approximately the same as or underestimates VE_NE_.

## 4. Discussion

Heterogeneity complicates the ability to accurately estimate the extent of vaccine-induced protection in a population, as well as if protection is truly waning or merely appears to be waning. While this has been extensively investigated for underlying frailty [4,5,6], the confounding effect of heterogeneity in vaccine protection has been less-thoroughly explored. In many cases [7,12,39], only all-or-nothing and leaky vaccines are investigated but we argue that these are edge cases that are wonderful for illustrating theory but are unlikely to accurately model real-world responses to vaccination. In particular, this study considers a much wider array of distributions and shows that the net effect of selection on these heterogeneities can cause either an increase or a decrease in mVE, with the effect given by concise and interpretable formulas.

We parameterized our model using data from epidemiological and immunological studies and also incorporated within-host modeling of the immune system and potential pathogens. We found that, within the estimated ranges, mVE is likely to be underestimated; however, the degree of underestimation is quite varied with heterogeneity in vaccine response, offsetting anywhere from less than 10% to greater than 100% (median of 29%) of the effect of heterogeneity in underlying susceptibility alone (Table S1 in the Supplementary Materials). Therefore, vaccine effectiveness estimates should be interpreted with caution, especially over time as the heterogeneities continue to accumulate differential outcomes. While mVE seems more likely to underestimate than overestimate VE_NE_, underestimation should not be assumed, as our range of plausible parameters includes cases without any underestimation.

Previous studies have used all-or-nothing or beta distributions to model vaccine-induced protection [7,12,39]. However, modeling waning with such distributions is not straightforward. Our technique of modeling the decay of immune responses (in our case, antibodies) at the individual level and converting these immune responses into individual level VE is more transparent and possibly easier to implement than modeling waning by, for example, shifting a beta distribution over time.

There are some important caveats to interpreting our results. Although Equations (2)–(4) give the effect of selection on the hazard rates and hazard ratios, the hazard rates and ratios can also be affected by regression towards (or away from) the mean. Regression towards the mean would tend to mitigate the effects of both heterogeneities. Secondly, in our simulations, heterogeneity in underlying susceptibility and heterogeneity in vaccine response are uncorrelated at baseline. Allowing for correlations permits for more diverse outcomes and affects not only waning but also the initial level of mVE. It should be noted that Equations (2)–(4) are valid even in the presence of such correlations. Extending our simulations to include such correlations and also regression towards or away from the mean is straightforward. In the current study, we focused only on the hazard ratio as a measure of VE. We did not consider the effect of seasonality, spatial structure, or epidemic waves. We also did not consider all possible combinations of distributions, but rather limited ourselves to those implied by data for acute respiratory infections.

## 5. Conclusions

We have brought together antibody waning, evolutionary theory, immune correlates of risk, vaccine-effectiveness estimation and within-host modeling in order to understand the effect of heterogeneity on the perceived waning of vaccine effectiveness. Further exploration of potential synergies between these areas of research may prove fruitful. For example, immune correlates of risk studies may be able to inform within-host modeling, and joint data analysis of correlates of risk, vaccine effectiveness over time and the waning of immune responses may allow for the more precise estimation and deconvolution of confounding factors.

Overall, we have given concise statistical formulas for understanding the effect of selection on mVE and have given estimates of the magnitude of this effect under a variety of situations based on both antibody and frailty data [10,38]. Our results suggest that VE_NE_ is likely but not certain to be higher than mVE due to variation at the individual level, and that the level of discrepancy is dependent on the specifics of the population and vaccine, meaning that a simple overall correction cannot be applied. Further exploration for how to correct for these factors statistically or via the study design is essential to more accurately understanding vaccine-induced protection.

## Data Availability

The code used to generate the simulations for this study as well as for its analysis can be found upon publication at the Zarnitsyna Lab Github (https://github.com/ZarnitsynaLab/ArielNikas-VEHeterogeneity).

## Supplementary Materials

The following supporting information can be downloaded at: https://www.mdpi.com/article/10.3390/vaccines11081312/s1, Supplementary Text S1.
