# Supplementary material for: Competing Heterogeneities in Vaccine Effectiveness Estimation
Source: Vaccines (Basel). 2023 Aug 1;11(8):1312. doi: 10.3390/vaccines11081312 (PMC10458793; doi:10.3390/vaccines11081312)
Supplement: Supplementary file 1 [file vaccines-11-01312-s001.zip › vaccines-2489092-supplementary.pdf]

Supplementary Text S1

Competing Heterogeneities in Vaccine Effectiveness Estimation

Ariel Nikas, Hasan Ahmed, and Veronika I. Zarnitsyna

1. Alternative Non-symmetric Beta Distributions

While the beta distributions considered in the main text were selected to reflect potential heterogeneity under vaccination, such that the vaccine either protected most people reasonably similarly (Beta(2, 2)) or gave a wide range of protection with some people responding strongly and others not (Beta(0.5, 0.5)), we also tested other distributions but they did not qualitatively alter our results. In Figure S1, we compare non-extreme, skewed beta distributions which, while they did both trend upwards, did not have large biases. However, changing these two skewed distributions to be more extreme (Figure S1 G, J), we see greater bias upwards as expected.

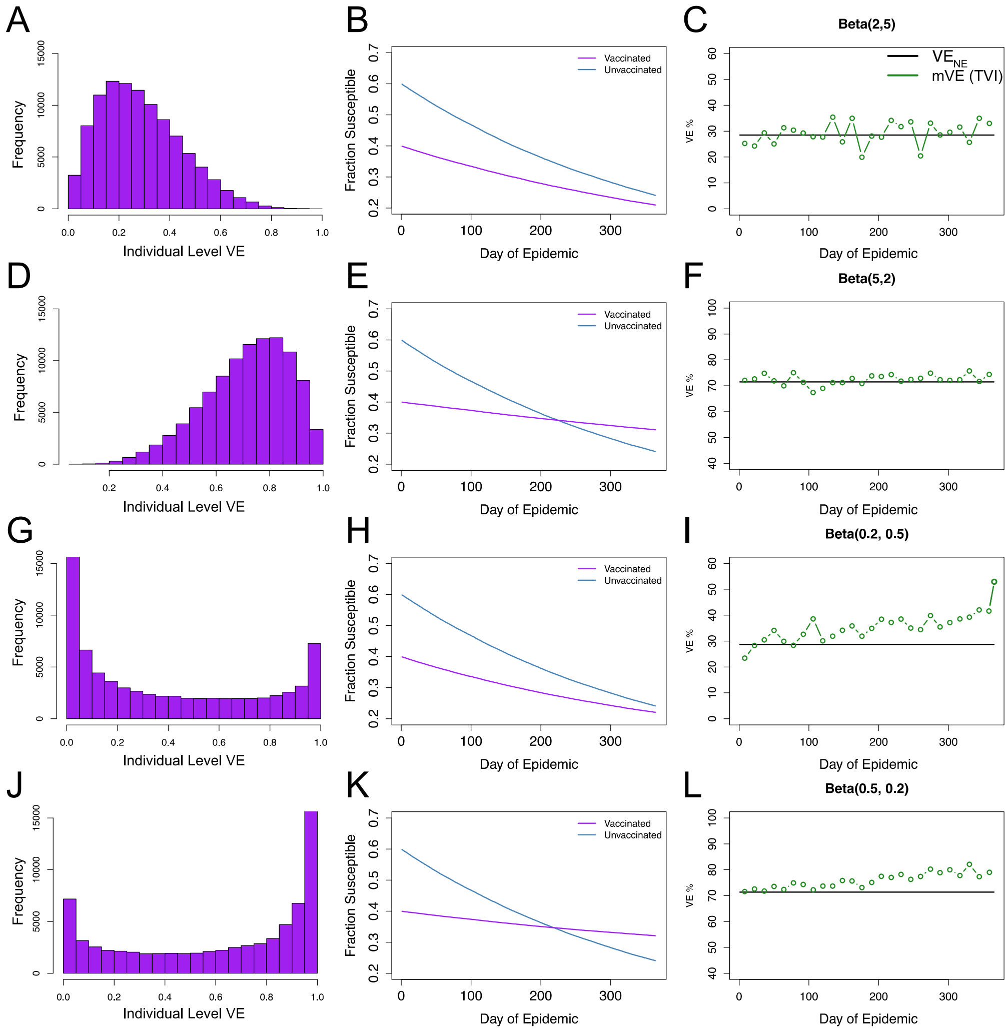

Figure S1. Non-symmetric beta distributions also bias upwards. Our results hold for other beta distribution shapes as well. In the first two rows we compare skewed but non-extreme distributions where in the first row the majority of the vaccinated population have little protection and in the second row the majority have high protection. We compare this to a more extreme version of each in the third and fourth rows. While the less extreme distributions remain near though slightly above VENE, the others increase more obviously with one increasing approximately 20%.

2. Starting Vaccine-induced Protection Influences the Total Amount of Perceived Waning

The initial level of protection from vaccination, VE(0), influences the amount of perceived waning observed. Extreme VE(0) show less observed change while middle values consistently showed the most. This pattern holds for both heterogeneity in vaccine response and heterogeneity in underlying frailty, as shown in the comparisons in Figures S2. Under no heterogeneity in susceptibility, leaky vaccination caused no more than 1% observed change in mVE in either direction. When α_γ is 1, change in mVE ranged from -7 to -17% while when α_γ is 2 the change in mVE ranged from -4 to -10% with the largest decreases being at the mid-range values for both. These changes are plotted explicitly in Figure S2.

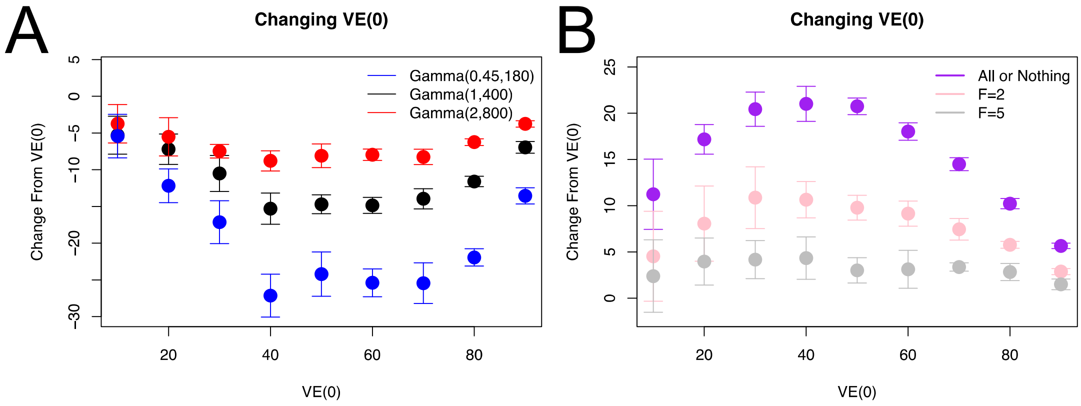

Figure S2. Effect of starting VE protection level. Different VE(0) values affect how much mVE can change. In Panel A, when there is no heterogeneity in individual level VE, regardless of chosen gamma distribution mid-range values of VE have the greatest capacity to change based on the difference from the initial value and the mean of the last five time points. In Panel B, if there is no heterogeneity in susceptibility but only in vaccine-induced protection as given by either a beta distribution or all-or-nothing protection, again midrange values have the largest capacity for change but this time bias upwards. For the beta distributions, $F=1+\alpha_\beta+\beta_\beta$ indicates the fold reduction in variance relative to all-or-nothing protection with the same mean. For VE = 50%, the F=2 point corresponds to the main text Figure 2F and the F=5 point corresponds to Figure 2C.

3. Predictions Not Necessarily Monotonic

While in the main text a variety of distributions were considered, we also tested additional distribution parameters for both the underlying heterogeneity in susceptibility and vaccine protection. Most of these combinations yielded monotonic predictions for change in VE; however, some, like the one pictured in Figure S3, can be non-monotonic. Here the predicted value decreases after a short period as can be seen by the dashed purple line appearing below the original value and then later increases past the original line around day 225 before dipping down and up yet again.

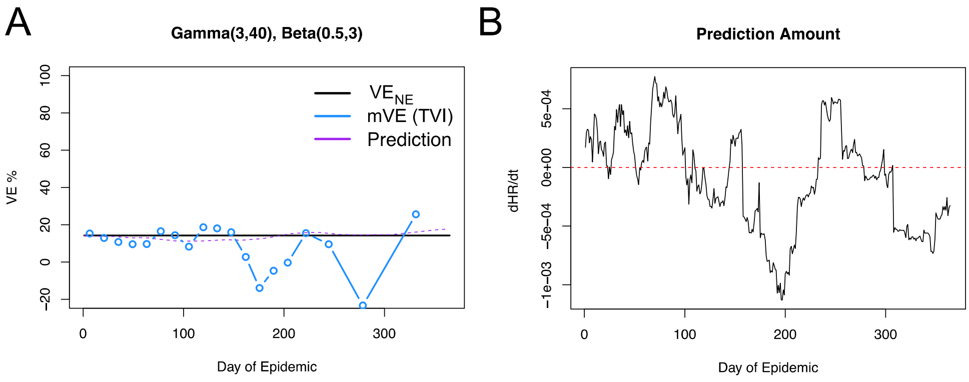

Figure S3. The observed and predicted vaccine effectiveness are not necessarily monotonic. While most parameter regimes considered yielded monotonic predictions for VE, that is not necessarily the case for any given parameter combination. Here we show an example where the predicted value both increases and decreases over time. This is especially evident in Panel B, as anytime the predicted change (black) crosses zero (red dashed), the direction of the change switches.

4. Unadjusted Antibody Exponent

In the main text, we explain our reasoning for increasing the antibody to VE conversion as HAI-specific titers are likely a lower bound for total antibody. We show results using this lower bound derived from [1] in Figure S4.

Here, instead of the main text conversion equation (Equation 7), we use

$$1 - VE_{NE} = \min[Ab^{-0.35}, 1]. \quad (1)$$

This exponent was approximated from the HAI titers found in [1]. This change did not qualitatively alter results; heterogeneity in vaccine protection continues to bias the VE estimate upwards while heterogeneity in the underlying susceptibility continues to bias the VE estimate downwards and in most scenarios will out-compete vaccine protection's bias.

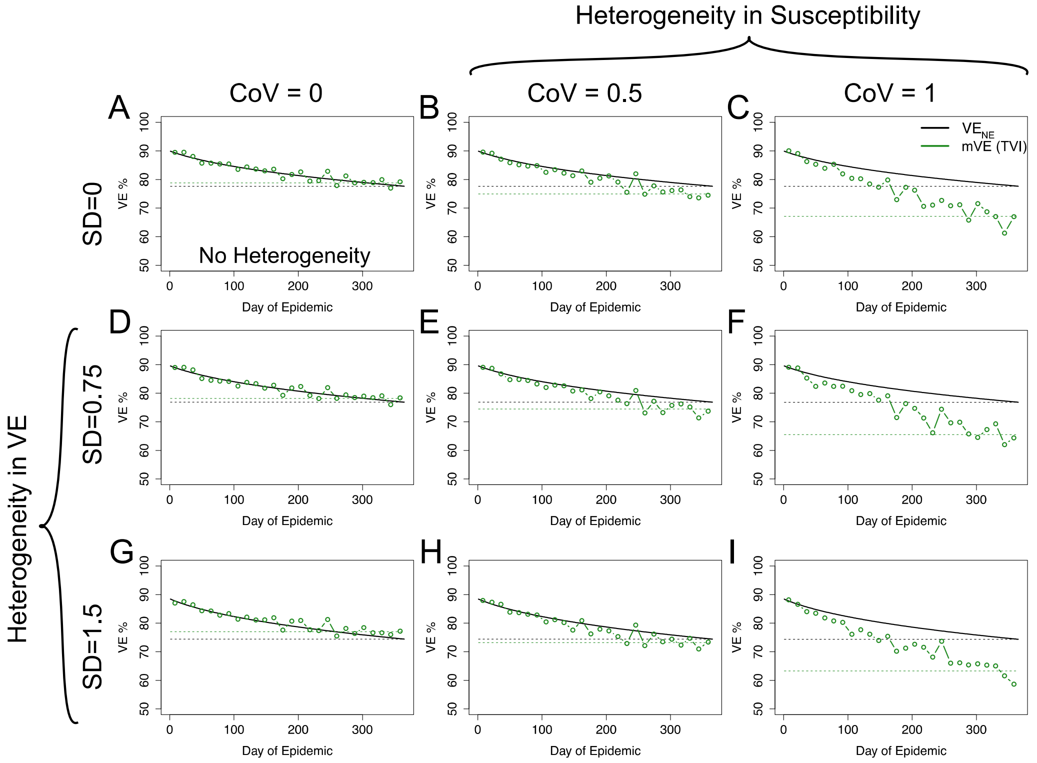

Figure S4. Unadjusted data estimate: overestimation of waning when frailty is added. Using the unadjusted power law exponent of -0.35 as estimated from [1] has similar behavior to the adjusted version seen in the main text. Again, increasing heterogeneity in underlying susceptibility (moving left to right) biases mVE downwards while increasing heterogeneity in vaccine-induced protection (moving top to bottom) biases mVE upwards. When these effects are mixed, as in Panels E, F, H, and I, the heterogeneities compete.

4. Within-Host Stochastic Model: Derivation

Here, we keep the same level of waning antibodies as given in Equation 6 of the main text, but we additionally consider the chance that an inoculum actually succeeds in causing infection. For clarity, we begin by defining the three reproductive numbers underpinning our model. We first define a cellular basic reproduction number R_0 which gives how

many new infected cells are produced from a single infected cell at the beginning of infection if no antibodies are present. This quantity can be estimated *in vitro*. We then define the reproductive number for the stochastic extinction model, R_s, to be the actual number of cells infected by any single infected cell in the presence of antibodies. This number is different for every infected cell and is selected from a distribution. The mean of the infected cells' R_s is referred to as R_0^*.

The probability that a single virion infects a cell is,

$$P_{inf} = \frac{r}{r+a+k\text{Ab}} \approx \frac{r}{a+k\text{Ab}}, \quad (2)$$

where r is the rate of infection, a is the rate of viral death, $k{\cdot}Ab$ is the rate of clearance by antibody, and $r \ll (a + k{\cdot}Ab)$. Assuming that a virion successfully infects a cell, we take the probability of early stochastic extinction to be

$$P_{SE} = min[1/R_0^*, 1], \quad (3)$$

as follows from the assumption that R_s, the actual number of cells infected by a given cell early in infection, is Gamma Poisson distributed with R_s ~ Poisson(mean=M) and M~Gamma($\alpha\gamma$=1, $\beta\gamma$=R_0^*-1). Additionally, we assume that R_0^* is proportional to the infectivity of the virus, so

$$R_0^* \approx R_0 \frac{a}{a+k\text{Ab}}, \quad (4)$$

where R_0 is the basic reproduction number of a cell in the absence of antibody. Building upon Equations S2 and S3, we then consider an inoculum with n virions where the probability of infection which escapes early stochastic extinction is

$$P_n = max\left[1 - \left(1 - \frac{r}{a+k\text{Ab}}\left(1 - \frac{a+k\text{Ab}}{aR_0}\right)\right)^n, 0\right]$$
$$\approx max\left[1 - exp\left(\frac{r}{a} \cdot n \cdot \left(\frac{-a}{a+k\text{Ab}} + \frac{1}{R_0}\right)\right), 0\right]. \quad (5)$$

This then becomes a hazard ratio HR_W by normalizing the above, yielding

$$HR_W = max\left[\frac{1-exp\left[\frac{m}{R_0} - \frac{m \cdot a}{a+k\cdot\text{Ab}}\right]}{1-exp\left[\frac{m}{R_0} - m\right]}, 0\right], \quad (6)$$

where we set $m = \frac{rn}{a}$.

We take the viral death rate to be a=10 in line with [2]. For the basic reproduction number in the absence of antibody, we use $R_0 = 10$ which is in the range estimated in [2].

For our combined parameter $m = \frac{rn}{a}$, we have additional considerations.

In the absence of antibody, the probability of infection taking hold is the denominator of Equation S6. Here, m in (0,1] corresponds to P_n of up to 59% per exposure in naive individuals and hence covers both small and moderately large exposures. As seen in Figure S5 changing m from near 0 to 1 does not result in large changes in the hazard ratio and therefore we chose m=0.5 as a representative for this range. If m is extremely large, this causes the hazard ratio to essentially become all-or-nothing.

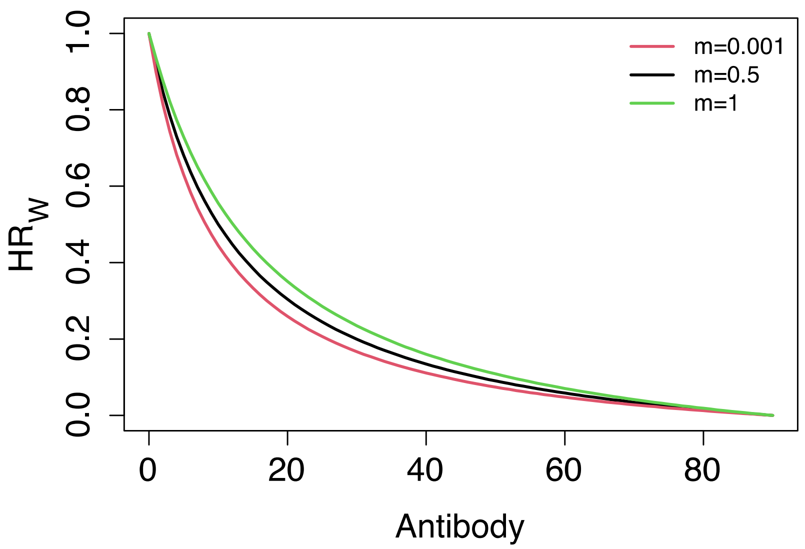

Figure S5. Effect of *m* on the hazard ratio. Hazard ratio results are not particularly sensitive to choice of *m* in (0,1] and thus a midrange value was selected (*m*=0.5).

5. Comparison of Risk-Correlate and Within-Host Stochastic Model Outcomes

The difference in end-season estimates from VE_{NE} for both the risk-correlate model and the within-host stochastic model are given in Table S1, below.

Table S1. Average of the difference between mVE and VE_{NE} over the last three time points. These values indicate the effect size of heterogeneity on mVE in our simulations. Heterogeneity in underlying susceptibility alone led to mVE underestimating VE_{NE} by 3.6%, 8%, 15.4%, and 22.9%. Adding heterogeneity in vaccine response offset anywhere from a negligible (<10%) to >100% (median: 29%) of the effect of heterogeneity in underlying susceptibility alone.

CoV	SD	Risk-Correlate	Within Host
0	0	0.45	0.6
0	0.75	1.6	2.9
0	1.5	3.9	6.9
0.5	0	-3.6	-8.0
0.5	0.75	-3.6	-5.0
0.5	1.5	2.0	2.0
1	0	-15.4	-22.9
1	0.75	-14.4	-18.2

1	1.5	-13.1	-12.0

References

1. Ng S, Fang VJ, Ip DK, Chan KH, Leung GM, Peiris JS, et al. Estimation of the association between antibody titers and protection against confirmed influenza virus infection in children. J Infect Dis. 2013;208(8):1320-4.

2. Ke R, Zitzmann C, Ho DD, Ribeiro RM, Perelson AS. In vivo kinetics of SARS-CoV-2 infection and its relationship with a person's infectiousness. Proc Natl Acad Sci U S A. 2021;118(49).
